# Dysregulated 14-3-3 Family in Peripheral Blood Leukocytes of Patients with Schizophrenia

**DOI:** 10.1038/srep23791

**Published:** 2016-03-31

**Authors:** Ying Qing, Liya Sun, Chao Yang, Jie Jiang, Xuhan Yang, Xiaowen Hu, Donghong Cui, Yifeng Xu, Lin He, Dongmei Han, Chunling Wan

**Affiliations:** 1 Shanghai Mental Health Center, Bio-X Institutes, Key Laboratory for the Genetics of Developmental and Neuropsychiatric Disorders (Ministry of Education), Key Laboratory of Translational Psychiatry, Shanghai Jiao Tong University, Shanghai 200030, China; 2 Instrumental Analysis Center, Shanghai Jiao Tong University, Shanghai 200240, China

## Abstract

The 14-3-3 family, which is composed of seven distinct members in humans, plays important roles in the cell cycle, apoptosis, synaptic plasticity and neuronal differentiation and migration. Previous genetic and post-mortem gene expression studies have linked this family to schizophrenia. However, the direction of gene expression changes in these studies has been inconsistent, and reports of 14-3-3 gene expression in living schizophrenic patients are still lacking. Here, we assessed 14-3-3 gene and protein expression levels in peripheral blood leukocytes from drug-naïve first-episode schizophrenic patients and matched controls. mRNA and protein expression levels were quantified by qRT-PCR and UPLC-MRM/MS, respectively. Expression analysis revealed four downregulated and one upregulated mRNA transcripts as well as five downregulated protein levels of 14-3-3 isoforms in schizophrenia. Moreover, significant positive correlations between 14-3-3 mRNA and protein expression levels were found in schizophrenia, and we also identified negative correlations between ε, θ and ζ isoform expression levels and positive symptoms of schizophrenia. Our results suggest that gene and protein expression levels for the 14-3-3 family are dysregulated in schizophrenia, perhaps owing to specific regulatory mechanisms, and we also suggest that expression of the 14-3-3ε, θ and ζ isoform genes could be useful indicators of disease severity.

Schizophrenia (SZ), a complex and debilitating mental disorder, affects more than 21 million people worldwide. The disorder generally occurs in late adolescence or early adulthood and usually continues throughout the life of the patient, creating a serious burden for family members and the healthcare system. SZ is characterized by profound disturbances in perception, thinking, emotion and behaviour. However, despite decades of research, the aetiology and pathophysiology of SZ remains elusive.

The 14-3-3 protein family is composed of 28–33 kDa acidic proteins that are found in all eukaryotic organisms. In humans, there are seven highly conserved members of this family, which are located on seven separate chromosomes. More specifically, 14-3-3β is encoded by the *YWHAB* gene on chromosome 20; 14-3-3ε is encoded by *YWHAE* on chromosome 17; 14-3-3γ is encoded by *YWHAG* on chromosome 7; 14-3-3η is encoded by *YWHAH* on chromosome 22; 14-3-3θ is encoded by *YWHAQ* on chromosome 2; 14-3-3ζ is encoded by *YWHAZ* on chromosome 8; and 14-3-3σ is encoded by *SFN* on chromosome 1. In general, the different isoforms self-assemble into homo- or heterodimers and interact with a diverse array of cellular proteins, with the exception of 14-3-3σ, which preferentially forms homodimers^1^. To date, several hundred distinct binding partners for the 14-3-3 proteins have been identified, representing 0.6% of the human proteome^2^. These 14-3-3 proteins function as molecular scaffolds by regulating the conformation of their binding partners through specific phospho-serine/phospho-threonine binding activities, and they have been implicated in cell signalling, gene transcription, metabolism, neurodevelopment, cell-division and apoptosis^3^,^4^,^5^,^6^.

14-3-3 proteins are abundantly expressed in the nervous system and play important roles in neuronal differentiation, migration and survival, synaptic plasticity, and ion channel regulation^7^. Over the past two decades, many genetic and gene/protein expression studies have linked the 14-3-3 family to SZ^8^,^9^,^10^,^11^,^12^,^13^,^14^. With respect to genetic studies, multiple members of this family are considered as candidate risk genes for SZ. 14-3-3η was the first member of this family to be identified, and it is located on chromosome 22q12.1-q13.1, a well-known region associated with SZ^11^,^15^,^16^. Linkage analyses have identified multiple single nucleotide polymorphisms (SNPs) in 14-3-3ε, 14-3-3η and 14-3-3ζ in several populations of patients with SZ^8^,^11^,^14^,^17^. With respect to gene/protein expression studies, post-mortem analyses of various SZ brain regions have shown decreased mRNA levels for six members of the 14-3-3 family (β, γ, ε, ζ, η and θ)^10^,^12^ and decreased protein levels for two 14-3-3 members (ε^18^,^19^ and ζ^20^,^21^,^22^). However, other studies have reported conflicting results, including increased 14-3-3ε protein expression in the dorsolateral prefrontal cortex^22^ and increased 14-3-3ζ protein expression in the prefrontal cortex^23^, anterior cingulate cortex^24^, insular cortex^25^ and mediodorsal thalamus^26^ in SZ patients.

Although brain tissue is perhaps the most relevant biological sample for aetiological studies of mental diseases such as SZ, considering that only post-mortem brain tissue is ethically and practically available, potential confounding variables, including pathological progression, antipsychotic medication, post-mortem changes and tissue storage time, must be taken into account when drawing conclusions from such studies. By contrast, studies in living patients using peripheral blood present several advantages and can complement and enrich results from post-mortem analyses of the central nervous system. First, peripheral blood is readily accessible, allowing for better control of the sample background. Second, several studies have indicated that gene expression in the brain and peripheral blood often show parallel responses^27^,^28^, or in other words, gene expression in the peripheral blood may be a useful reference for gene expression in the brain, at least in cases when the genes of interest are expressed in both tissues. Third, a number of studies have suggested that irregular peripheral functions, such as an impaired immune activity, are involved in the onset of SZ^29^,^30^,^31^. Therefore, peripheral changes that affect the aetiology of SZ could be studied in the blood to complement findings in the brain.

In this study, to determine the gene and protein expression profiles of 14-3-3 family members in SZ, we recruited drug-naïve first-episode patients with SZ and matched controls. We quantified the mRNA and protein levels of 14-3-3 isoforms in peripheral blood leukocytes (PBLs), determined the correlations between mRNA expression and protein abundance for each member of the 14-3-3 family, and, finally, analysed the associations between 14-3-3 gene/protein expression and SZ symptoms.

## Materials and Methods

### Subject selection

We recruited twenty-four antipsychotic drug-naïve first-episode patients who were diagnosed as schizophrenic, according to the Diagnostic and Statistical Manual of Mental Disorders, fourth edition (DSM-IV® Dissociative Disorders [SCID-D-R]), as well as twenty-four healthy controls with no family history of SZ or detectable medical, psychiatric or neurological problems from the Second Xiangya Hospital of Central South University in Changsha Province, China (Table 1). Healthy controls were matched for age, gender and body mass index. All participants were non-smokers. Positive and negative syndrome scale (PANSS) ratings were completed through face-to-face interviews with trained raters to determine psychiatric symptom severity. The study was conducted in accordance with the Declaration of Helsinki, and informed consent was obtained after an explanation of the study was provided. All experiments were performed in accordance with approved guidelines, and this study was approved by the Ethics Committee of Bio-X Institutes, Shanghai Jiao Tong University.

### Isolation of leukocyte total RNA and protein from whole blood samples

Blood was collected in 5 mL EDTA-treated tubes early in the morning following an overnight fast (~10–12 h). Two millilitres of blood was mixed with 10 mL precooled red blood lysis buffer (Tiangen, Beijing, China) to lyse the erythrocytes. After a 10 min incubation on ice, the mixture was centrifuged at 2,000 rpm at 4 °C for 10 min. The supernatant was removed, and the precipitated pellet was rinsed again with lysis buffer. After the supernatant was carefully decanted, 1 mL TRIzol**®** reagent (Invitrogen, Carlsbad, CA, USA) was added to the clear pellet of leukocytes to extract total RNA and protein, according to the manufacturer’s instructions. The RNA pellet and protein pellet were stored at −80 °C until use. The quality and quantity of the isolated RNA were assessed by electrophoresis (Agilent 2100 Bioanalyzer, Santa Clara, CA, USA) and spectrophotometry (NanoDrop, Rockland, DE, USA), respectively.

### Quantitative real-time polymerase chain reaction (qRT-PCR)

One microgram of qualified total RNA from each sample was reverse transcribed into cDNA in a 20 μL reaction using the PrimeScript® RT reagent kit with gDNA Eraser (TaKaRa, Dalian, China). 14-3-3 expression was measured using qRT-PCR with an ABI PRISM® 7900HT system (Applied Biosystems, Foster City, CA, USA) using a TaqMan® Low Density Array (TLDA) with factory-designed assays for all 14-3-3 genes (SFN, Hs00968567_s1; YWHAB, Hs00793604_m1; YWHAE, Hs00356749_g1; YWHAG, Hs00705917_s1; YWHAH, Hs00607046_m1; YWHAQ, Hs00863277_g1; YWHAZ, Hs03044281_g1). Each assay included specific primers and TaqMan probes, and all reactions were performed in duplicate using *ACTB* (β-actin, Hs99999903_m1) as an internal reference control. Amplification plots and Ct values for the seven target and single reference genes are shown in Supplementary Figures S1–S8 and Supplementary Table S1.

### Trypsin digestion

The protein pellet was solubilized in 100 μL reduction solution (6 M guanidinium and 50 mM NH_4_HCO_3_) and then centrifuged at 14,000 × *g* at 4 °C for 10 min to remove insoluble materials. The supernatant was transferred to a new 1.5 mL microfuge tube, and the protein concentration was determined using the Bradford method. Fifty micrograms of protein was transferred to a new 1.5 mL microfuge tube and chemically reduced with 20 mM dithiothreitol at 56 °C for 1 h, followed by alkylation with 90 mM iodoacetamide at 25 °C for 40 min in the dark. The protein solution was then transferred to a new 10 kDa ultrafiltration tube (Sartorius, Goettingen, Germany) and centrifuged at 14,000 × *g* at 4 °C for 20 min to eliminate contaminants less than 10 kDa in size. Then, 100 μL 50 mM NH_4_HCO_3_ was added to the ultrafiltration tube, and the mixture was centrifuged at 14,000 × *g* at 4 °C for 20 min; this wash step was repeated 3 times. Sequencing-grade porcine trypsin (Promega, Madison, WI, USA) was added to the final digestion mixture at a 1:50 enzyme-to-substrate ratio (w/w). The mixture was incubated overnight (16 h) at 37 °C and then centrifuged at 14,000 × *g* at 4 °C for 20 min. Fifty microlitres of 25 mM NH_4_HCO_3_ was added to the ultrafiltration tube for elution, and the sample was centrifuged at 14,000 × *g* at 4 °C for 20 min to collect the peptide solution. The digestion was stopped by acidification with formic acid to a final pH < 3. All peptide samples were dehydrated using a vacuum centrifuge and dissolved in 0.1% formic acid before mass spectrometry analysis.

### Ultra-performance liquid chromatography-multiple reaction monitoring/mass spectrometry (UPLC-MRM/MS)

Samples were analyzed on a Q-Trap 5500 (AB Sciex, Framingham, MA, USA) mass spectrometer coupled with an Acquity UPLC system (Waters, Millford, MA, USA). An Acquity HSS T3 column (100 Å, 1.8 μm, 1.0 × 50 mm, Waters) was used to separate 5 μL samples, according to the gradient listed in Supplementary Table S2, at a flow rate of 0.2 mL/min. Pools of twenty-four schizophrenic samples and twenty-four healthy control samples were used for quality control (QC) to determine the stability of the instruments, and a single process of each sample was analysed. A coefficient of variation (CV) of QC < 15% indicated qualified data.

### Statistical analysis

Relative quantification of gene expression was assessed using the comparative threshold cycle (Ct) method. ΔCT was calculated as the Ct difference between the target gene and the housekeeping gene (*ACTB*). The relative amount of mRNA for each target gene in each sample was calculated as 2^−ΔCt^. If the discrepancy between the two repeated measurements was larger than 0.5 Ct, the corresponding average Ct was annotated as a missing value. Missing values, representing 1.6% of the total data, were imputed with the mean values to fully utilize available information and facilitate the following analysis. The relative quantification of each peptide was obtained by summing the peak areas of the corresponding transitions. The relative quantification of each protein in each sample was expressed as the mean of the two peptides per protein. The normality of the variable distribution was assessed using the Kolmogorov-Smirnov (KS) test. The homogeneity of the variances was evaluated using an F test. Outliers that were more than 3 standard deviations from the mean were not included in the following analysis. Student’s *t*-tests for equal or unequal variances were performed to determine statistically significant changes in mRNA or protein abundances. *p*-values resulting from the *t*-test were adjusted with the false discovery rate (FDR). The conclusions of the UPLC-MRM/MS results derived from the *t*-test were verified by comparing the results to the output of R using the MSStats program^32^, which relies on linear mixed effect models. Correlations between mRNA levels and protein levels were tested using Pearson’s correlation analysis. Spearman’s correlation analysis was used to determine the correlation between relative mRNA/protein expression and the PANSS scores. Statistical analyses were conducted using the R software program (http://www.r-project.org/) and GraphPad Prism 5 (GraphPad Software, Inc., San Diego, CA, USA). A value of *p* < 0.05 was considered to represent a statistically significant difference.

## Results

### Quantification of 14-3-3 mRNA transcripts by qRT-PCR

**Figure 1:**
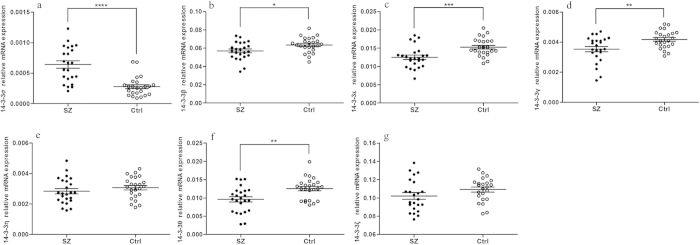
Relative mRNA expression levels of the 14-3-3 isoforms in schizophrenic (SZ) patients and healthy control (Ctrl) subjects. (**a**) 14-3-3σ. (**b**) 14-3-3β. (**c**) 14-3-3ε. (**d**) 14-3-3γ. (**e**) 14-3-3η. (**f**) 14-3-3θ. (**g**) 14-3-3ζ. Horizontal and vertical lines indicate the mean ± S.E.M. values. *p < 0.05, **p < 0.01, ***p < 0.001, ****p < 0.0001.

To analyse 14-3-3 isoform mRNA levels in SZ patients, we isolated PBL RNA for analysis by qRT-PCR. In general, the isolated RNA samples were of very high quality. Twenty-nine out of forty-eight RNA samples had RIN values greater than or equal to 9, seventeen samples had RIN values greater than 8 but less than 9, and only two RNA samples had RIN values greater than 7 but less than 8 (7.8 and 7.6). Expression of 14-3-3σ was significantly increased in SZ patients compared with control subjects (2.29-fold, *p* < 0.0001, Fig. 1a). Expression of 14-3-3β (−1.12-fold, *p* = 0.016, Fig. 1b), 14–3–3ε (−1.22-fold, *p* < 0.001, Fig. 1c), 14–3–3γ (−1.18-fold, *p* = 0.001, Fig. 1d) and 14–3–3θ (−1.30-fold, *p* = 0.002, Fig. 1f) was significantly decreased in SZ patients. After FDR correction of the expression data for the seven isoforms, the differences observed between SZ patients and controls remained statistically significant. Expression of 14-3-3η (Fig. 1e) and 14-3-3ζ (Fig. 1g) was not significantly different in SZ patients.

### Quantification of 14-3-3 family proteins by UPLC-MRM/MS

Due to the very low abundance of the 14-3-3σ protein in PBLs, six of the seven 14-3-3 isoforms were investigated using UPLC-MRM/MS. Their amino acid sequences, m/z values of the precursor ions, retention time and fragment ions used for quantification are summarized in Supplementary Table S3.

**Figure 2:**
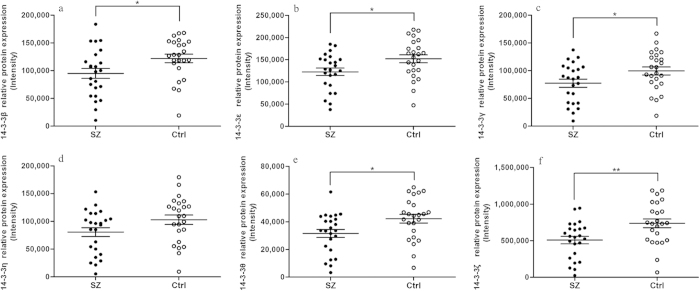
Relative protein expression levels of the 14-3-3 isoforms in schizophrenic (SZ) patients and healthy control (Ctrl) subjects. (**a**) 14-3-3β. (**b**) 14-3-3ε. (**c**) 14-3-3γ. (**d**) 14-3-3η. (**e**) 14-3-3θ. (**f**) 14-3-3ζ. Horizontal and vertical lines indicate the mean ± S.E.M. values. *p < 0.05, **p < 0.01.

Five of the analysed isoforms showed decreased protein levels in SZ patients, similar to what was observed for the mRNAs. Expression of the 14-3-3β protein was significantly reduced in SZ patients (−1.28-fold, *p* = 0.026, Fig. 2a). The decrease in 14-3-3ε protein levels (−1.24-fold, *p* = 0.021, Fig. 2b) was comparable to the decrease in 14-3-3ε mRNA levels (−1.22-fold, *p* < 0.001, Fig. 1c). Additionally, the 14-3-3γ (−1.29-fold, *p* = 0.038, Fig. 2c), 14–3–3θ (−1.34-fold, *p* = 0.018, Fig. 2e) and 14–3–3ζ (−1.45-fold, *p* = 0.005, Fig. 2f) proteins were all significantly decreased in SZ patients. All decreases were also significant after FDR correction. By contrast, expression of the 14-3-3η protein was not significantly different, similar to what was found for its transcript levels (Fig. 2d).

### Correlations between transcript levels and protein expression for the 14-3-3 family members

**Figure 3:**
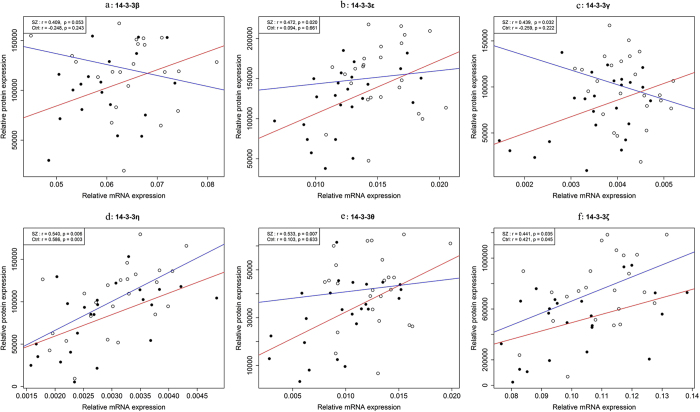
Correlations between mRNA and protein levels in six members of the 14-3-3 family. (**a**) 14-3-3β. (**b**) 14-3-3ε. (**c**) 14-3-3γ. (**d**) 14-3-3η. (**e**) 14-3-3θ. (**f**) 14-3-3ζ. Pearson correlation coefficients (r) and *p*-values (p) are shown in the upper left corner of each plot. Solid circles and red lines represent data from the schizophrenia group (SZ); circles and blue lines represent data from the control group (Ctrl).

We analysed the correlations between 14-3-3 isoform transcript levels and protein expression using Pearson correlations in SZ patients and control subjects. Interestingly, the correlations between mRNA transcript levels and protein expression differed between the two groups (Fig. 3). In SZ patients, we found a significant positive correlation between mRNA levels and protein expression for 5 isoforms (Fig. 3b–f) as well as a positive correlation trend for 14-3-3β (Fig. 3a). However, in control subjects, only 14-3-3η (Fig. 3d) and 14-3-3ζ (Fig. 3f) transcript levels showed a significant positive correlation with protein expression. Significant correlations between mRNA levels and protein expression were not observed for the other four isoforms in control subjects.

### Associations between 14-3-3 family members and symptoms in SZ patients

**Figure 4:**
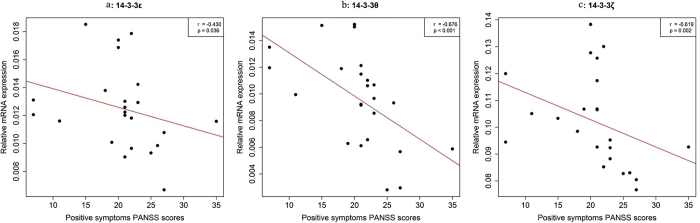
Associations between the expression of three 14-3-3 family members and positive symptoms PANSS scores. (**a**) 14-3-3ε. (**b**) 14-3-3θ. (**c**) 14-3-3ζ. Spearman correlation coefficients (r) and *p*-values (p) are shown in the upper right corner of each plot.

PANSS is a medical scale used for typological and dimensional assessment of symptom severity in patients with SZ^33^. There are thirty items rated from 1 to 7 on the PANSS, which are classified into 3 dimensions: positive symptoms, negative symptoms and general symptoms. We compared the PANSS scores (PANSS total score, positive score, negative score and general score) (Table 1) with mRNA levels (Fig. 1) and protein expression (Fig. 2) using Spearman’s correlation analysis. Interestingly, we found a significant negative correlation between mRNA levels for 14-3-3ε and positive symptoms PANSS scores (Fig. 4a). Additionally, 14-3-3θ (Fig. 4b) and 14-3-3ζ (Fig. 4c) mRNA levels were negatively correlated with positive symptoms PANSS scores as well. Therefore, for the first time, we link 14-3-3 isoform expression levels to disease severity in SZ.

## Discussion

The 14-3-3 family is a ubiquitous eukaryotic adaptor protein family that regulates a wide variety of cellular processes by altering the activity, phosphorylation state, localization and stability of various protein partners^7^,^34^,^35^. Extensive genetic linkage and association studies have suggested that 14-3-3 family members play a role in SZ^8^,^9^,^11^,^13^,^36^. In particular, a number of studies have examined 14-3-3 family gene/protein expression in the brains of patients with SZ, but conflicting results have been obtained (Table 2).

In this study, we used PBLs from drug-naïve first-episode patients with SZ and matched controls to explore the role of 14-3-3 family members in SZ. To the best of our knowledge, we are the first to show dysregulated expression patterns for the 14-3-3 family in living SZ patients. qRT-PCR analysis revealed that mRNA expression levels for 14-3-3β, ε, γ and θ were downregulated in the SZ patients, consistent with a previous study using post-mortem brain samples^10^. We also detected increased expression for 14-3-3σ, which was a novel result. Using UPLC-MRM/MS analysis, we also examined the relative protein levels of the 14-3-3 isoforms in the same cohort. Strikingly, we found that five of the seven isoforms (β, ε, γ, θ and ζ) showed significantly reduced protein levels in the SZ samples compared with controls.

The 14-3-3 proteins activate tryptophan hydroxylase, a rate-limiting enzyme involved in serotonin synthesis. Considering the “serotonin hypothesis” for the aetiology of SZ, expression changes for the 14-3-3 family could contribute to SZ by disturbing the serotonin pathway – a possibility that requires further exploration. Additionally, studies have reported that 14-3-3ε levels are decreased in the synapses in NR1 KD mice^37^, which has been linked to reduced dopamine synthesis through the regulation of tyrosine hydroxylase levels and activity^38^. Interestingly, 14-3-3ζ also regulates tyrosine hydroxylase^39^, and 14-3-3γ promotes tyrosine hydroxylase localization to the membrane to preserve enzymatic activity^40^. Tyrosine hydroxylase is a rate-limiting enzyme in catecholamine (dopamine, adrenaline and noradrenaline) synthesis, and the catecholamine neurotransmitter dopamine plays an important role in SZ. Therefore, these previous studies, along with our results, suggest that the 14-3-3 family members may play a specific role in neurotransmission regulation by altering tyrosine hydroxylase localization and function in SZ. Furthermore, a recent study found that inhibition of 14-3-3 proteins in mice leads to behavioural abnormalities related to SZ^41^. Our findings support the hypothesis that decreased expression of the 14-3-3 isoforms is an indicator of SZ. Moreover, the 14-3-3 family also functions in numerous cell processes in leukocytes. For example, in activated T cells, both α_L_β_2_ integrin and filamin are binding partners of 14-3-3, and these interactions can mediate different T cell functions^42^. In B cells, 14-3-3 proteins function as scaffolds for class switch DNA recombination (CSR)^43^, and they regulate B cell homeostasis by maintaining FOXO1 levels^44^. In monocytes, degradation of 14-3-3ζ following metabolic stress promotes monocyte migration^45^. Thus, the changes in 14-3-3 gene/protein expression observed in this study might also undermine immune cell function in SZ. Finally, the epigenetic changes at the 14-3-3σ locus were reported to be an indicator of chronic inflammation^46^, a condition that has been linked to SZ^47^. Thus, based on our results, it seems worthwhile to investigate epigenetic modifications of the 14-3-3σ gene in SZ.

We also assessed the correlations between 14-3-3 gene and protein expression in SZ. In the present study, we found that mRNA and protein levels were more highly correlated in SZ patients than in control subjects (Fig. 3). In other words, for most 14-3-3 family members, mRNA and protein levels changed in unison in SZ patients but not in control subjects, suggesting that 14-3-3 protein levels in SZ are tightly regulated by the downregulation of de novo synthesis. However, for 14-3-3η and ζ, gene expression and protein levels were highly correlated in controls as well, and they were the only 14-3-3 isoforms that did not show significantly different mRNA expression profiles in the SZ patients. Therefore, it appears that these isoforms have distinct regulatory patterns compared with the other family members and maintain their normal expression patterns under disease conditions. As no details concerning the regulation or co-regulation status of these seven 14-3-3 isoforms in SZ have yet been reported, these results require further exploration.

We also identified associations between gene expression and disease severity in SZ. We found that expression of the 14-3-3ε, θ and ζ genes were negatively correlated with positive SZ symptoms, a finding that could have important ramifications of patients’ conditions. Therefore, reduced mRNA expression for the 14-3-3ε, θ, and ζ isoforms could be useful as indicators of disease severity in SZ patients.

To summarize, we report for the first time the differential expression of specific 14-3-3 isoforms, at both the gene and protein levels, in a cohort of living SZ patients. We found that (1) SZ patients have altered 14-3-3 isoform gene and protein expression patterns, which may be characteristic of SZ; (2) there may be specific regulatory mechanisms for the expression of certain 14-3-3 family members in SZ, which requires further investigation; and (3) gene expression levels for certain 14-3-3 family isoforms might be useful indicators of disease severity in SZ. However, these results will require further validation in larger cohorts. In addition, we recommend further study of the epigenetic modifications and molecular functions of the 14-3-3 family members in the context of SZ.

## Additional Information

**How to cite this article**: Qing, Y. *et al.* Dysregulated 14-3-3 Family in Peripheral Blood Leukocytes of Patients with Schizophrenia. *Sci. Rep.* **6**, 23791; doi: 10.1038/srep23791 (2016).

## Supplementary Material

Supplementary Information

## Figures and Tables

**Table 1:** Demographic data of SZ patients and healthy control subjects.

	SZ (n = 24)	Control (n = 24)
Gender (M/F)	12/12	12/12
Age	25.2 ± 0.7	24.7 ± 0.3
Waist (cm)	77.4 ± 1.8	75.9 ± 1.7
Height (cm)	164.0 ± 1.4	163.8 ± 2.0
Weight (kg)	56.8 ± 2.2	56.9 ± 1.8
BMI (kg m^−2^)	21.0 ± 0.6	21.1 ± 0.3
PANSS Total Score	77.0 ± 2.2	–
PANSS Positive Score	20.7 ± 1.3	–
PANSS Negative Score	21.9 ± 1.6	–
PANSS General Score	34.4 ± 1.2	–

Abbreviations: SZ, schizophrenia; M, male; F, female; BMI, body mass index; PANSS, positive and negative syndrome scale. Data (age, waist, height, weight, BMI, PANSS total score, PANSS positive score, PANSS negative score, PANSS general score) are shown as the mean ± S.E.M. values.

**Table 2:** Altered 14-3-3 isoform expression reported in other studies.

Isoform	Direction of Differential Expression	Type of Analysis	Tissue	mRNA or Protein Expression	References
β	↓	Microarray & ISH	PFC	mRNA	10
	↑	WB	PFC	Protein	23
	↑	2-DE	ACC	Protein	24
ε	↓	Microarray	PFC	mRNA	10
	↓	2-DE	CC	Protein	18
	↓	ProteinChip	dlPFC	Protein	19
	↑/−	2D-DIGE/ELISA	dlPFC	Protein	22
γ	↓	ISH	PFC	mRNA	10
	↓	2-DE	CC	Protein	18
	↓	Shotgun	ATL	Protein	20
	↓	2-DE	ACC	Protein	24
η	↓	Microarray & ISH	PFC	mRNA	10
	↓	Neuroarray	Cerebellum	mRNA	12
	↓	Shotgun	ATL	Protein	20
θ	↓	Microarray	PFC	mRNA	10
	↓	2D-DIGE	IC	Protein	25
ζ	↓	Microarray & ISH	PFC	mRNA	10
	↓	2-DE	CC	Protein	18
	↓	Shotgun	ATL	Protein	20
	↓	2D-DIGE	Thalamus	Protein	21
	↓	2D-DIGE/ELISA	dlPFC	Protein	22
	↑	WB	PFC	Protein	23
	↑	2-DE	ACC	Protein	24
	↑	2D-DIGE	IC	Protein	25
	↑/↓	iTRAQ/2-DE	MDT	Protein	26

PFC, prefrontal cortex; ACC, anterior cingulate cortex; CC, corpus callosum; dlPFC, dorsolateral prefrontal cortex; ATL, anterior temporal lobe; IC, insular cortex; MDT, mediodorsal thalamus.
