# Supplementary material for: Dysregulated 14-3-3 Family in Peripheral Blood Leukocytes of Patients with Schizophrenia
Source: Sci Rep. 2016 Mar 31;6:23791. doi: 10.1038/srep23791 (PMC4814835; doi:10.1038/srep23791)
Supplement: Supplementary Information [file srep23791-s1.pdf]

Title

Dysregulated 14-3-3 Family in Peripheral Blood Leukocytes of Patients with Schizophrenia

Authors

Ying Qing[†], Liya Sun[†], Chao Yang, Jie Jiang, Xuhan Yang, Xiaowen Hu, Donghong Cui,

Yifeng Xu, Lin He, Dongmei Han, Chunling Wan[*]

[†] These authors contributed equally to this work.

[*] Corresponding author

Supplementary Figure legends

Supplementary Figure S1 14-3-3σ (SFN) amplification plot in qRT-PCR.

Supplementary Figure S2 14-3-3β (YWHAB) amplification plot in qRT-PCR.

Supplementary Figure S3 14-3-3ε (YWHAE) amplification plot in qRT-PCR.

Supplementary Figure S4 14-3-3γ (YWHAG) amplification plot in qRT-PCR.

Supplementary Figure S5 14-3-3η (YWHAH) amplification plot in qRT-PCR.

Supplementary Figure S6 14-3-3θ (YWHAQ) amplification plot in qRT-PCR.

Supplementary Figure S7 14-3-3ζ (YWHAZ) amplification plot in qRT-PCR.

Supplementary Figure S8 β-Actin (ACTB) amplification plot in qRT-PCR.

Supplementary Figure S1

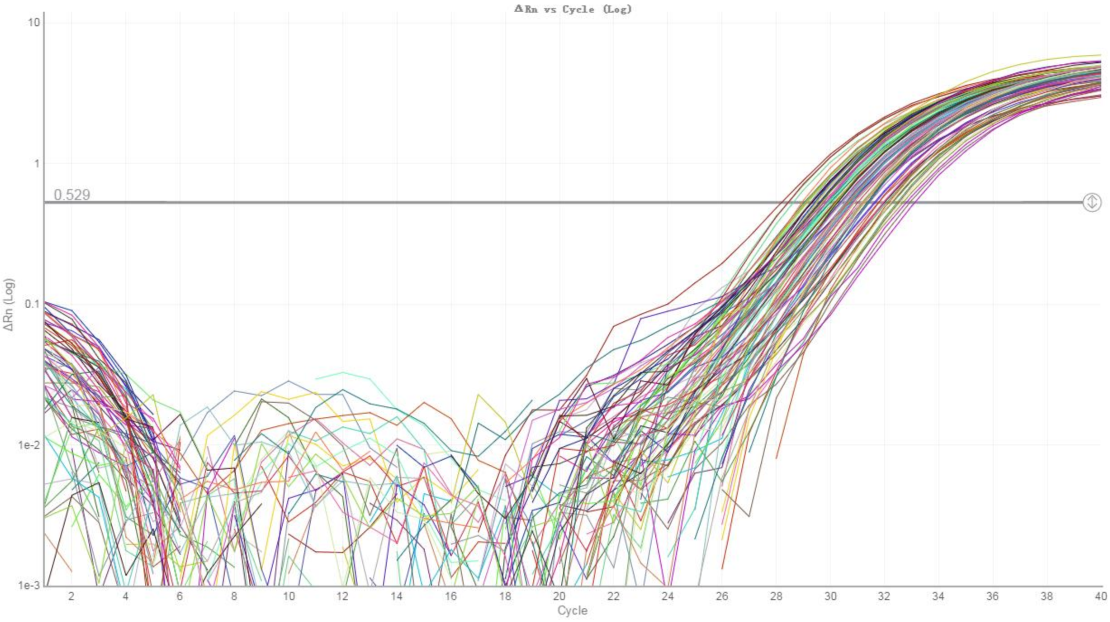

Supplementary Figure S2

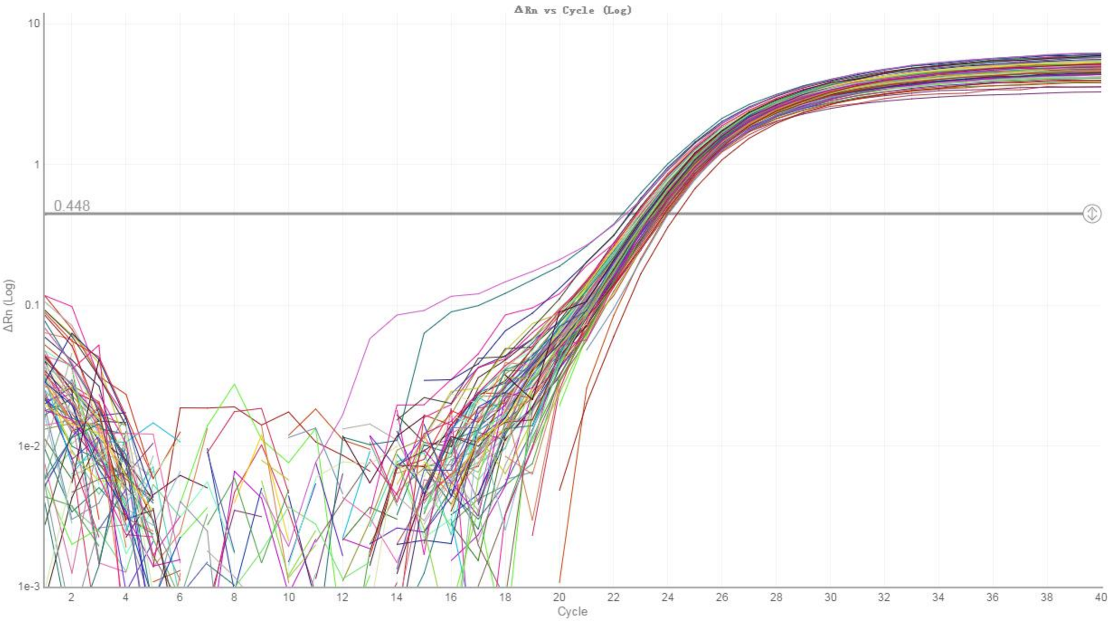

Supplementary Figure S3

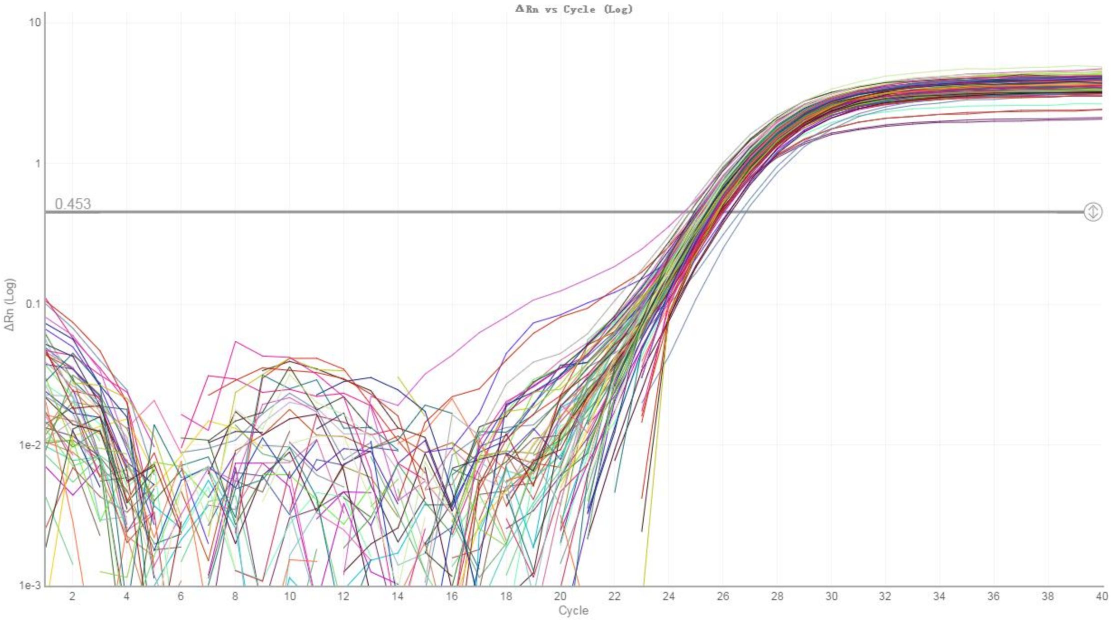

Supplementary Figure S4

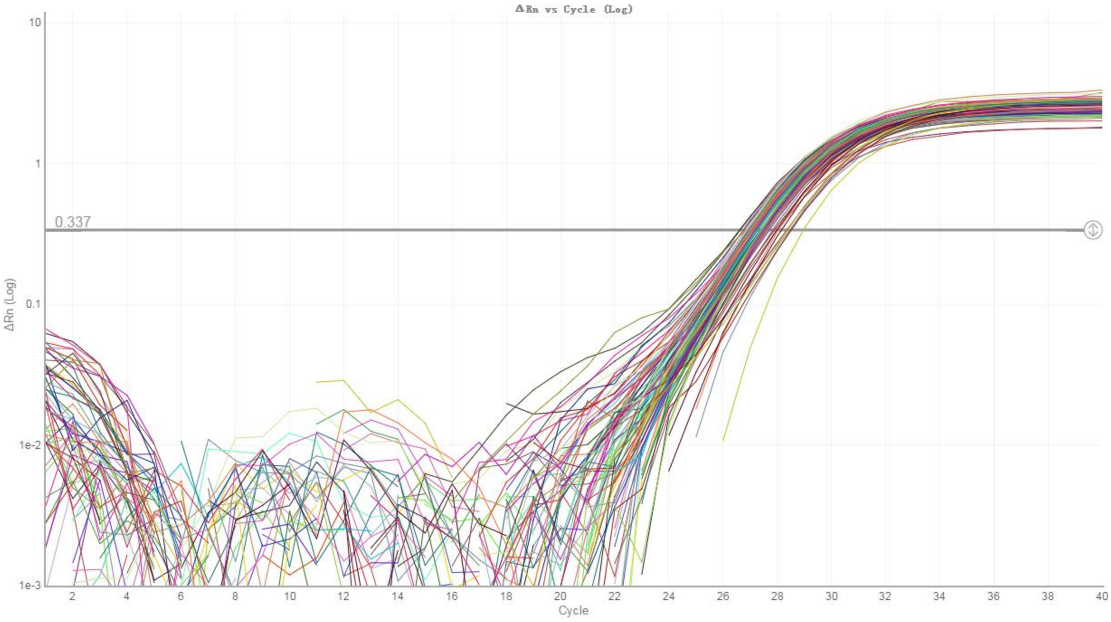

Supplementary Figure S5

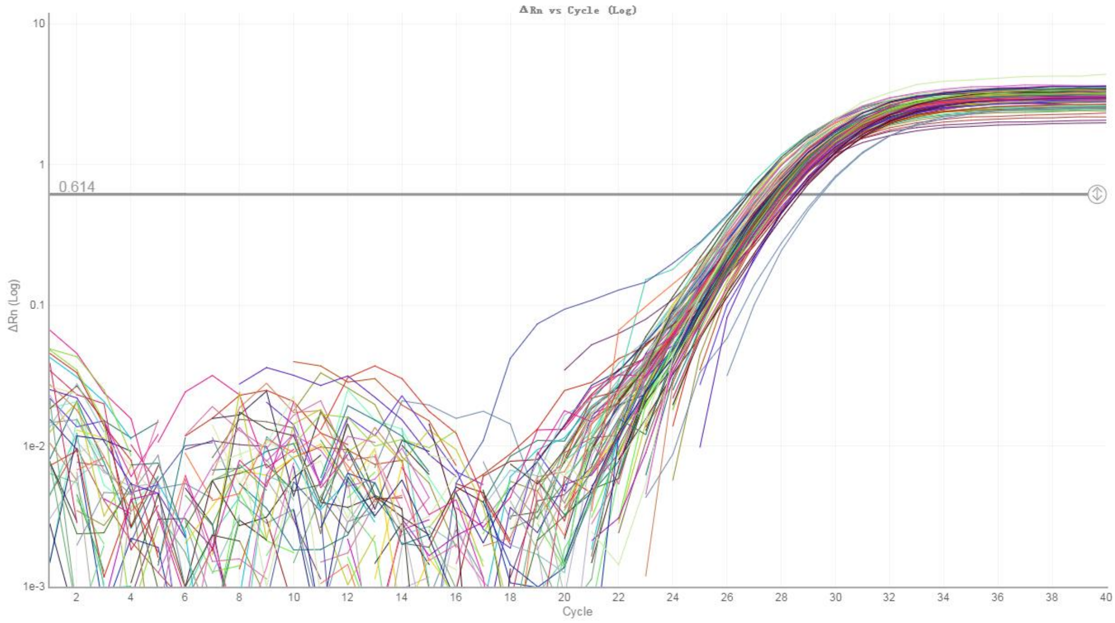

Supplementary Figure S6

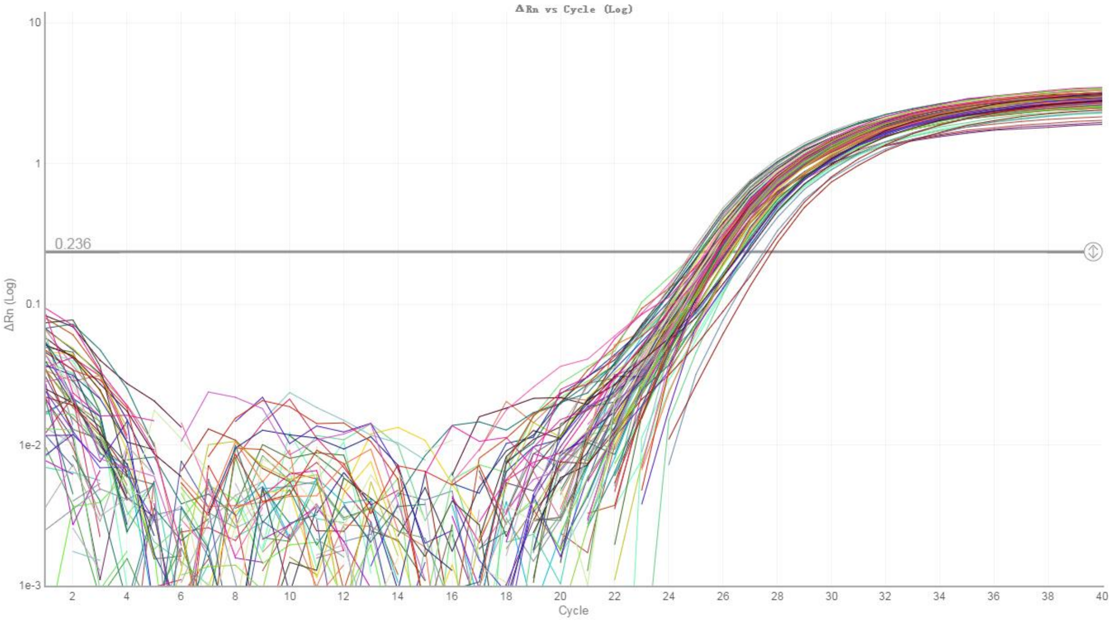

Supplementary Figure S7

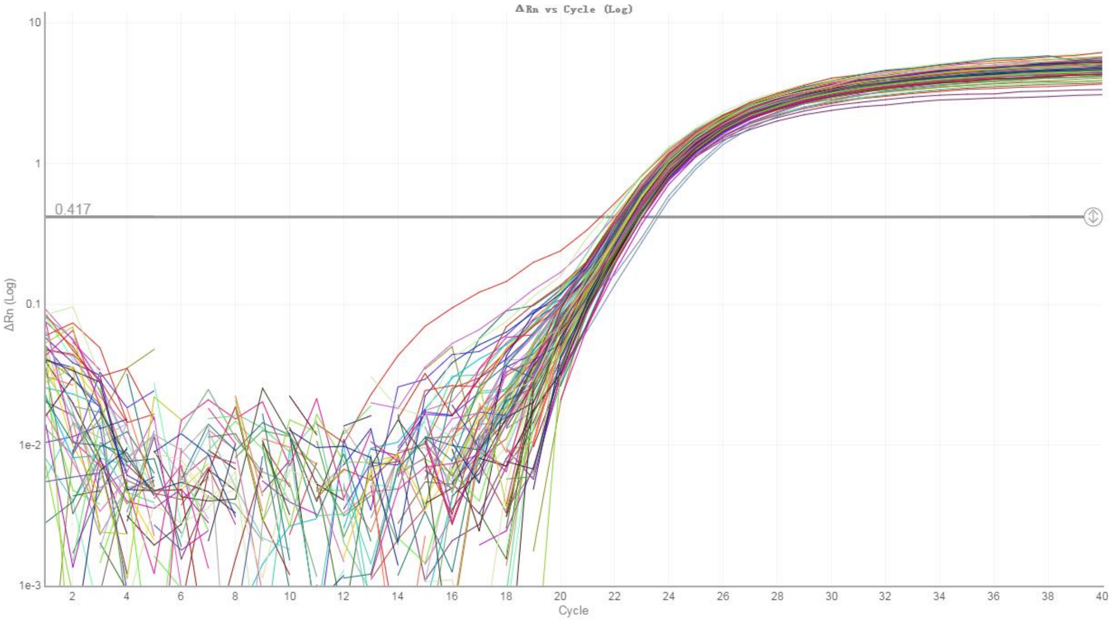

Supplementary Figure S8

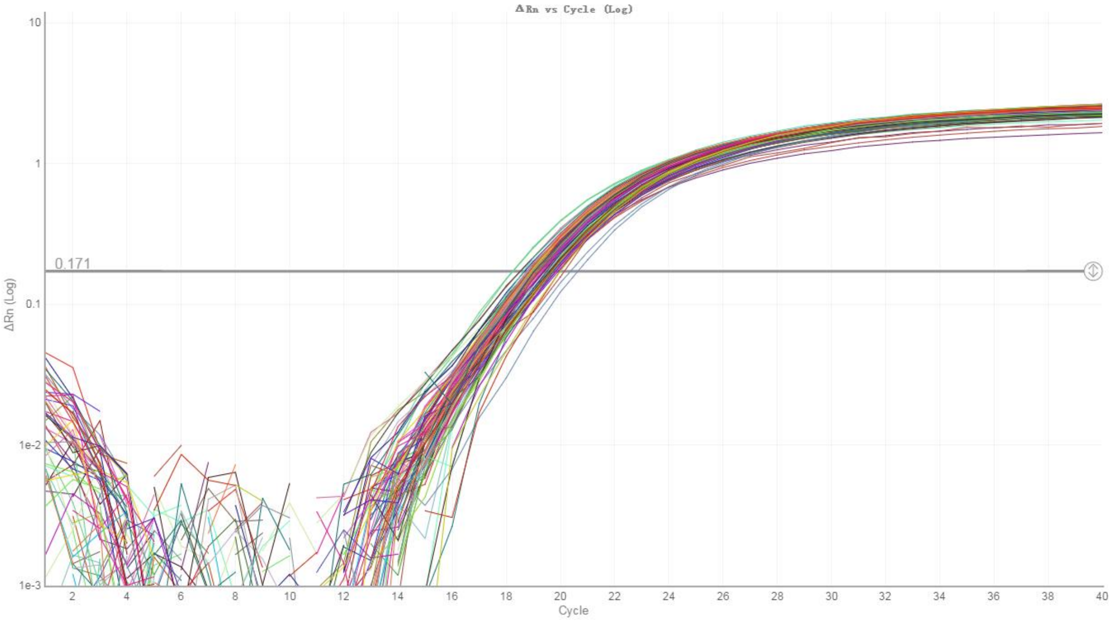

Supplementary Table S1 Ct values of target and reference genes in qRT-PCR.

Gene Symbol	T/R	Ct Values (n = 48)		
SFN	T	30.62	±	1.13
YWHAB	T	23.33	±	0.29
YWHAE	T	25.48	±	0.36
YWHAG	T	27.32	±	0.38
YWHAH	T	27.73	±	0.49
YWHAQ	T	25.85	±	0.56
YWHAZ	T	22.51	±	0.31
ACTB	R	19.27	±	0.40

Abbreviations: T, target gene; R, reference gene. Ct values are mean ± S.D. values.

Supplementary Table S2 HPLC gradient for quantitation of the peptides

	Time (min)				
	0	1	9	15	17
Mobile phase A (%)	100	100	85	70	60
Mobile phase B (%)	0	0	15	30	40

Mobile phase B: 0.1% formic acid in acetonitrile.

Mobile phase A: 0.1% formic acid in H_2O.

Supplementary Table S3 Peptides used for MRM

Gene Symbol	Protein	Peptides	Precursor ions (m/z)	Retention Time (min)	Fragment Ion
YWHAB	14-3-3β	AVTEQGHELSNEER	533.585	5.38	y7, y6, y5, y4, y2
		YLSEVASGDNK	591.7855	6.15	y9, y8, y7, y5, y4
YWHAE	14-3-3ε	EAAENSLVAYK	597.8037	7.70	y7, y6, y5, y4, y3, y2
		YLAEFATGNDR	628.7989	8.79	y8, y7, y6, y5, y4, b4
YWHAG	14-3-3γ	AYSEAHEISK	378.8524	4.86	y6, y5, y4, b4
		YLAEVATGEK	540.7822	7.19	y9, y8, y7, y6, y5, y4
YWHAH	14-3-3η	YLAEVASGEK	533.7744	6.77	y9, y8, y7, y6, y5, y4
		AVTELNEPLSNEDR	793.8865	8.46	y8, y7, y5, y4, b4, b5
YWHAQ	14-3-3θ	SICTTVLELLDK	696.3762	14.58	y7, y6, y5, y3, b4
		YDDMATCMK	567.7169	6.75	y7, y6, y5, y4, y3
YWHAZ	14-3-3ζ	SVTEQGAELSNEER	774.8604	6.59	y8, y7, y6, y5, y4, y2
		FLIPNASQAESK	652.8459	9.64	y9, y8, y7, y6, y5, y4
